# Genetic characterisation of *Cryptosporidium* spp., *Encephalitozoon* spp., and *Enterocytozoon bieneusi* in European brown hare (*Lepus europaeus*) in Central Europe

**DOI:** 10.1051/parasite/2026042

**Published:** 2026-08-06

**Authors:** Alena Konvalinová, Marie Křesťanová, Nikola Holubová, Jana Fenclová, Marta Kicia, Matúš Rajský, Pavel Beran, Bohumil Sak, Martin Kváč

**Affiliations:** 1 Faculty of Agriculture and Technology, University of South Bohemia in České Budějovice Czechia; 2 Czech Union of Nature Conservationists Animal Rescue Center Vlašim Czechia; 3 Institute of Parasitology Biology Centre of the Academy of Sciences of the Czech Republic, v.v.i České Budějovice Czechia; 4 Faculty of Science, University of South Bohemia in České Budějovice Czechia; 5 Department of Biology and Medical Parasitology, Wroclaw Medical University Wroclaw Poland; 6 National Agricultural and Food Centre Lužianky Slovakia; 7 Faculty of Forestry, Technical University in Zvolen Zvolen Slovakia

**Keywords:** PCR, Hare, Genotyping, Geographic distribution, Infection intensity, *Giardia intestinalis*

## Abstract

The European brown hare (*Lepus europaeus*) is an ecologically important small game mammal whose populations have declined in many European countries. A total of 370 faecal samples collected between 2022 and 2024 from hares in the Czechia and Slovakia were molecularly screened for the presence of *Cryptosporidium* spp. (18S rRNA, *gp60*), *Giardia intestinalis* (*TPI*), *Encephalitozoon* spp., and *Enterocytozoon bieneusi* (ITS). qPCR analysis confirmed the presence of *Cryptosporidium* spp. (*n* = 15; 4.1%; CI: 2.5–6.6%), *Encephalitozoon* spp. (*n* = 34; 9.2%; CI: 6.7–12.6%), and *E. bieneusi* (*n* = 16; 4.3%; CI: 2.6–6.9%). Sequence analyses revealed the presence of *C. parvum* (*n* = 2), *C. sciurinum* (*n* = 3), *C. ubiquitum* (*n* = 5), and *Cryptosporidium* sp. deer mouse genotype IV (*n* = 5). At the *gp60* locus, *C. parvum* belonged to subtypes IIaA15G1R1 and IIaA23G1R1, *C. sciurinum* to subtypes VIIIcA10G2R1 (*n* = 2) and VIIIbA11G1R1 (*n* = 1), and *C. ubiquitum* to subtypes XIIa (*n* = 3) and XIId (*n* = 2). Five *E. bieneusi* genotypes were identified: D (*n* = 8), CHALT1 (*n* = 1), WildBoar3 (*n* = 1), CHN-F1 (*n* = 2), and C (*n* = 4). *Encephalitozoon* sequencing revealed *E. cuniculi* genotypes I (*n* = 6), II (*n* = 26), and III (*n* = 1), and *E. hellem* (*n* = 1). *Giardia intestinalis* was not detected. *Cryptosporidium* sp. deer mouse genotype IV is reported here for the first time in Europe.

## Abstract (fr)

Le lièvre d'Europe (*Lepus europaeus*) est un petit mammifère de gibier, écologiquement important, dont les populations ont décliné dans de nombreux pays européens. Au total, 370 échantillons de matières fécales prélevés entre 2022 et 2024 sur des lièvres en République tchèque et en Slovaquie ont été analysés par des méthodes moléculaires pour détecter la présence de *Cryptosporidium* spp. (ARNr 18S, *gp60*), *Giardia intestinalis* (*TPI*), *Encephalitozoon* spp. et *Enterocytozoon bieneusi* (ITS). L'analyse par qPCR a confirmé la présence de *Cryptosporidium* spp. (*n* = 15 ; 4,1 % ; IC : 2,5–6,6 %), *Encephalitozoon* spp. (*n* = 34 ; 9,2 % ; IC : 6,7–12,6 %) et *E. bieneusi* (*n* = 16 ; 4,3 % ; IC : 2,6–6,9 %). Les analyses de séquences ont révélé la présence de *C. parvum* (*n* = 2), *C. sciurinum* (*n* = 3), *C. ubiquitum* (*n* = 5) et *Cryptosporidium* sp. génotype IV « deer mouse » (*n* = 5). Au niveau du locus *gp60*, *C. parvum* appartenait aux sous-types IIaA15G1R1 et IIaA23G1R1, *C. sciurinum* aux sous-types VIIIcA10G2R1 (*n* = 2) et VIIIbA11G1R1 (*n* = 1), et *C. ubiquitum* aux sous-types XIIa (*n* = 3) et XIId (*n* = 2). Cinq génotypes d’*E. bieneusi* ont été identifiés : D (*n* = 8), CHALT1 (*n* = 1), WildBoar3 (*n* = 1), CHN-F1 (*n* = 2) et C (*n* = 4). Le séquençage d’*Encephalitozoon* a révélé les génotypes I (*n* = 6), II (*n* = 26) et III (*n* = 1) d’*E. cuniculi,* ainsi qu’*E. hellem* (*n* = 1). *Giardia intestinalis* n'a pas été détecté. Le génotype IV « deer mouse » de *Cryptosporidium* sp. est signalé ici pour la première fois en Europe.

## Introduction

The European brown hare (*Lepus europaeus*), originally native to the Eurasian steppes, now inhabits many countries worldwide and is an important small game species. In Europe, it mainly occupies agricultural landscapes, where it shares habitats with humans, livestock, and a wide range of wild animal species [14, 40, 41]. This ecological overlap creates opportunities for the transmission of environmentally transmitted pathogens, and the hare can serve as a natural reservoir. While information on the occurrence of *Toxoplasma gondii*, *Neospora caninum*, *Sarcocystis*, *Eimeria*, and larval stages of cestodes and nematodes in European brown hares is relatively abundant [12, 20, 52, 58], data on several other potentially pathogenic and zoonotic parasites, such as *Cryptosporidium* spp., microsporidia, and *Giardia intestinalis*, remain limited and are entirely absent in some regions.

In Czechia and Slovakia, molecular surveys have documented the occurrence of enteric microeukaryotes including *T. gondii*, *N. caninum*, *Sarcocystis*, *Eimeria*, *Cryptosporidium* spp., *Giardia intestinalis*, *Encephalitozoon* spp., and *Enterocytozoon bieneusi* in a variety of free-living wildlife hosts, including insectivores, bats, rodents, carnivores, ungulates or birds [2–4, 33, 36, 39, 43, 59]. Several of the detected species and genotypes have been recognized as zoonotic and may contribute to environmental transmission cycles. Despite the increasing knowledge of these pathogens in wildlife populations of Central Europe, information on their occurrence and genetic diversity in lagomorphs, particularly in the European brown hare, remains very limited.

*Cryptosporidium* is a genus of obligate parasitic apicomplexan protozoa that infects a wide range of vertebrates, including mammals, birds, reptiles, fish, and amphibians. With the exception of *C. parvum* and *C. ubiquitum*, which infect a broad range of mammalian hosts, and *C. baileyi* and *C. meleagridis*, which infect numerous avian species, most *Cryptosporidium* species show narrow host specificity [27, 45]. Nevertheless, more than twenty *Cryptosporidium* species and genotypes are considered to pose a potential infection risk to humans [37, 55].

*Giardia intestinalis* is an obligate intestinal parasite of humans and many animal species and consists of eight genetically distinct assemblages (A–H) that differ in their host ranges. While assemblages A and B have low host specificity and are associated with zoonotic potential, the other assemblages are predominantly host-specific [15, 48].

Of the more than 1,200 microsporidian species described to date, infections in mammals are primarily caused by *Enterocytozoon bieneusi* and members of the genus *Encephalitozoon* (*E. intestinalis*, *E. cuniculi*, and *E. hellem*). These species infect a wide range of mammalian hosts, including livestock, wildlife, companion animals, and humans, and most of their genotypes show broad host specificity with significant zoonotic potential [49]. Within the genus *Encephalitozoon*, four genotypes of *E. cuniculi* (I–IV), six genotypes of *E. hellem* (1A, 1B, 1C, 2A, 2B and 2C), and a single genotype of *E. intestinalis* have been distinguished based on sequence polymorphisms within the internal transcribed spacer (ITS) region of the rRNA gene and additionally analysing the polar tube protein gene. Although *E. cuniculi* genotypes were originally designated as rabbit (genotype I), mouse (genotype II), dog (genotype III), and human (genotype IV) strains, they exhibit low host specificity and have been detected in a wide range of mammalian hosts. Similarly, although *E. hellem* is most commonly associated with avian hosts, its genotypes have also been reported in mammals [35, 49, 61]. *Enterocytozoon bieneusi* exhibits considerably greater genetic diversity. To date, more than 500 genotypes have been described based on sequence polymorphisms within the ITS region of the rRNA gene. Phylogenetic analyses have grouped these genotypes into at least 15 distinct genetic groups. Genotypes belonging to groups 1 and 2 are considered to have the greatest zoonotic significance, as they have been detected in a broad range of hosts, including humans, livestock, companion animals, and wildlife, whereas the remaining groups are generally regarded as host-adapted and exhibit a narrower host range [30, 51].

Compared with other lagomorphs, particularly rabbits, knowledge about the occurrence of these parasites in hares is limited [16, 21, 46]. Only a few studies have investigated the presence of *Cryptosporidium* in hares, and most of them reported negative findings, likely due to inappropriate diagnostic methodologies or the low number of animals examined. A similar situation applies to *Giardia* and microsporidia [3, 6, 10, 14, 33, 38, 56].

The aim of the present study is to address the existing knowledge gaps regarding the geographical distribution and genetic variability of *Cryptosporidium*, *Giardia*, and microsporidia in European brown hares in Czechia and Slovakia, using molecular genotyping approaches and qPCR quantification of infection.

## Material and methods

### Ethics approval

Hares admitted to wildlife rescue centres were handled in accordance with Act No. 114/1992 Coll. on Nature and Landscape Protection (Czechia). Hunted animals were legally harvested in accordance with Act No. 449/2001 Coll., Low on Hunting (Czechia), and Act No. 274/2009 Coll. on Hunting and on the Amendment of Certain Acts, as amended (Slovakia). Faecal samples were collected as part of routine parasitological examinations, and only faecal material was subsequently provided for scientific purposes. No animals were captured, handled, or subjected to any experimental procedures specifically for this study, in accordance with Act No. 246/1992 Coll. on the Protection of Animals against Cruelty (Czechia). The use of the collected material for research purposes was approved by the Animal Welfare Committee of the Biology Centre, Czech Academy of Sciences.

### Area and specimens studied

**Figure 1:**
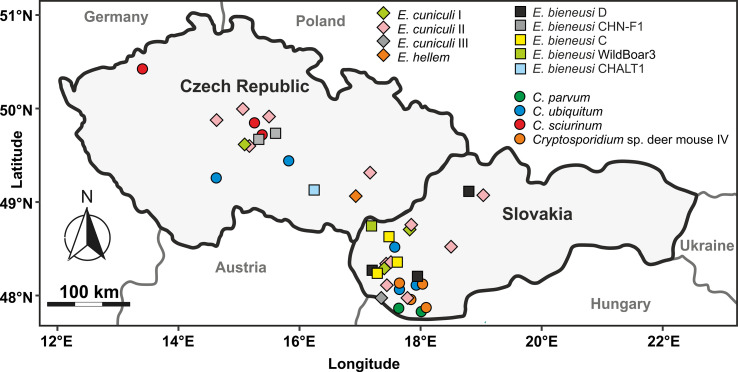
Map of the geographical distribution of *Cryptosporidium* spp., *Encephalitozoon* spp., and *E. bieneusi* in European brown hares (*Lepus europaeus*) in Czechia and Slovakia.

A cross-sectional molecular epidemiological study was conducted in Czechia and Slovakia between 2022 and 2024. Samples were collected from several localities in Czechia and Slovakia, representing the typical habitat of the European brown hare (Fig. 1). Czech samples originated from two main landscape types: the lowland agricultural region of the Elbe basin (180–250 m a.s.l.) and the more elevated, heterogeneous agricultural landscapes of Central Bohemia and the Bohemian-Moravian Highlands (300–600 m a.s.l.). Slovak sampling sites were mainly in the lowland and hilly agricultural regions of southwestern Slovakia (approximately 100–300 m a.s.l.). In all regions, habitats consisted mainly of arable land interspersed with grasslands, field margins, hedgerows, and woodland patches. Faecal samples were collected from European brown hares (*Lepus europaeus*) that were temporarily kept in wildlife rescue centres or were hunted. Faecal samples from hares admitted to rescue centres were collected immediately after arrival and always within 24 h before transfer to holding facilities. In hunted hares, faecal samples were collected directly from the large intestine. Each faecal sample was placed in a sterile plastic vial labelled with the sample ID and stored at 4–8 °C without fixative until laboratory processing within two weeks of collection. During handling in rescue centres or at the time of hunting, the general health status of each animal was assessed, including body condition and faecal consistency. Detailed information on sampling localities, the number of samples collected at each locality, and all available metadata for individual animals, including age and origin, are in Supplementary Table S1.

### Molecular analyses

Total genomic DNA (gDNA) was extracted from 200 mg of faeces using the Exgene™ Stool DNA mini kit (GeneAll Biotechnology Co. Ltd., Seoul, South Korea), following the manufacturer’s instructions with slight modification. Specifically, sterile 0.5 mm glass beads were added to the microtubes up to the 100 μL mark, followed by the addition of 10 sterile zirconia beads (2 mm in diameter; Next Advance, Troy, NY, USA) to each tube containing the sample and lysis buffer. The samples were then homogenised for 60 s at 5.5 m/s using a FastPrep^®^-24 device (MP Biomedicals, Santa Ana, CA, USA) [50]. The purified DNA was stored at −20 °C before being used for PCR. The positivity of samples for the presence of specific DNA of *Cryptosporidium* spp., *Giardia intestinalis*, *Encephalitozoon* spp., and *E. bieneusi* was tested using qPCR. Parasite infection intensity was expressed as the number of *Cryptosporidium* spp. oocysts (oocysts per gram – OPG), *E. bieneusi* and *Encephalitozoon* spp. spores (spores per gram – SPG), and *G. intestinalis* cysts (cyst per gram – CPG) per gram of the faeces. For detection and quantification of *E. bieneusi*, a commercial qPCR kit YSLqP-IC-E.biene-100 was used according to the manufacturer’s recommendations (YouSeq Ltd., Winchester, UK). *Cryptosporidium* spp., *G. intestinalis*, and *Encephalitozoon* spp. were quantified using a TaqMan-based qPCR assay and previously published protocols [18, 25, 60]. Briefly, each 20 μL reaction contained 2 μL gDNA, 400 nM forward and reverse primers, 200 nM 5′ FAM and 3′ BHQ1-labelled TaqMan probe and 10 μL Luna Universal Probe qPCR master Mix (New England BioLabs Inc., Ipswich, MA, USA). A list of all primers and probes used in this study is given in Supplementary Table S2. A standard (calibration) curve was constructed using 10^7^ isolated *Cryptosporidium parvum* oocysts, *G. intestinalis* assemblage D cysts, and *E. intestinalis* spores, which were subjected to a series of tenfold serial dilutions (10^1^–10^7^) to determine assay sensitivity and efficiency.

Samples that tested positive by qPCR were genotyped using nested PCR targeting a fragment of the small subunit ribosomal RNA (18S rRNA) gene for *Cryptosporidium*, the triosephosphate isomerase (*TPI*) gene for *Giardia intestinalis*, and the internal transcribed spacer (ITS) region of the small ribosomal subunit for *Encephalitozoon* spp. and *E. bieneusi*, following previously published protocols [9, 13, 24, 57, 62]. To investigate the genetic diversity among *Cryptosporidium* spp. isolates, additional PCR assays were conducted targeting the 60-kDa glycoprotein gene (*gp60*) in 18S rDNA-positive sample. For *gp60* subtyping, *C. sciurinum*-positive samples were analysed using the protocol described by Alves *et al.* [1], while *C. ubiquitum*-positive samples were analysed according to Li *et al.* [29]. The PCR reaction was carried out in a total volume of 30 μL. The reaction mixture contained 2 μL of gDNA as template, or 2 μL of the primary PCR product in case of a secondary reaction, 200 μM each of deoxynucleoside triphosphate (dNTP), 1× PCR buffer (DreamTaq™ Green Buffer, Thermo Fisher Scientific, Waltham, MA, USA), 3.0 mM MgCl_2_, 1 U of Taq polymerase (Thermo Fisher Scientific), 10 μg of bovine serum albumin (BSA) and 200 nM of each primer. *Cryptosporidium hominis*, *G. intestinalis* assemblage D, *E. intestinalis*, and *E. bieneusi* genotype PtEbIX DNA and molecular grade water were used as positive and negative controls, respectively. Secondary PCR amplicons were visualised on 1.5% agarose gels (SERVA Agarose for DNA Electrophoresis, SERVA Electrophoresis GmbH, Heidelberg, Germany) stained with ethidium bromide and purified using a GenElute Gel Extraction Kit (Sigma-Aldrich, St. Louis, MO, USA) prior to Sanger sequencing performed by a commercial company (SeqMe s.r.o, Dobříš, Czechia). Each positive sample was independently sequenced twice using both forward and reverse primers (bidirectional sequencing), and consensus sequences were generated from the resulting chromatograms.

### Phylogenetic and statistical analyses

Nucleotide sequences obtained for each gene were edited and processed in ChromasPro v2.4.1 (Technelysium Pty Ltd., South Brisbane, QLD, Australia). All chromatograms were manually inspected to assess sequence quality and identify potential ambiguous positions. Sequence alignments were generated using the MAFFT online platform (version 7; [http://mafft.cbrc.jp/alignment/software/]) with the automatic alignment mode option, comparing both study sequences and GenBank references. Phylogenetic reconstruction and model selection were performed in MEGA X using the maximum likelihood (ML) approach. The Bayesian Information Criterion (BIC) was applied to determine the best-fitting nucleotide and protein substitution models, and node reliability was evaluated by performing 1,000 bootstrap replicates [17, 26]. Resulting trees were exported from MEGA X and manually refined using CorelDRAW X7. Sequences obtained in this study were deposited in GenBank under the following accession numbers: for 18S rDNA [PX629147–PX629150], for *gp60* [PX619180–PX619185], and for ITS [PX627779–PX627782 and PX627774–PX627778]. Prevalence values of detected parasites were calculated with 95% confidence intervals (95% CI) using the Wilson Score Interval in R version 4.2.2. All analyses were conducted in R (version 4.2.2) with a significance threshold of *α* = 0.05.

## Results

A total of 370 faecal samples collected from European brown hares were analysed, including 130 samples from Czechia and 240 from Slovakia. qPCR analysis confirmed the presence of *Cryptosporidium* spp. (*n* = 15; 4.1%; CI: 2.5–6.6%), *Encephalitozoon* spp. (*n* = 34; 9.2%; CI: 6.7–12.6%), and *Enterocytozoon bieneusi* (*n* = 16; 4.3%; CI: 2.6–6.9%), with infection intensities ranging from 1.0 × 10^2^ to 9.8 × 10^3^ for *Cryptosporidium* spp., from 0.9 × 10^2^ to 6.4 × 10^2^ for *E. bieneusi*, and from 1.5 × 10^2^ to 7.6 × 10^3^ for *Encephalitozoon* spp. (Table 1). No samples tested positive for *Giardia intestinalis*. In subsequent nested PCR using genus-specific primers, all qPCR-positive samples were successfully amplified and sequenced by Sanger sequencing. Analysis of the 18S rRNA gene sequences of *Cryptosporidium* spp. revealed the presence of *C. parvum* (*n* = 2), *C. sciurinum* (*n* = 3), *C. ubiquitum* (*n* = 5), and *Cryptosporidium* sp. deer mouse genotype IV (*n* = 5). All sequences within each species or genotype were identical and showed 100% similarity to the corresponding sequences deposited in the GenBank database. Manual inspection of all chromatograms revealed no ambiguous nucleotide positions or consistent double peaks indicating mixed infections.

**Table 1:** Occurrence, infection intensity, and genotyping of *Cryptosporidium* spp., *Encephalitozoon* spp., and *Enterocytozoon bieneusi* in European brown hare (*Lepus europaeus*) in the Czech Republic (CZE) and Slovakia (SVK). Infection intensity is expressed as the number of oocysts (OPG) and spores per gram of faeces (SPG).

Country	Locality	ID sample	*Cryptosporidium* spp.		*Encephalitozoon* spp.		*E. bieneusi*	
			Genotyping	OPG	Genotyping	SPG	Genotyping	SPG
CZE	Bollinka-Loreta	53700	–	–	*E. cuniculi* II	2.1 × 10^2^	–	–
	Čestín	53709	–	–	–	–	CHN-F1	1.3 × 10^2^
	Domašín	53677	*C. sciurinum* VIIIb	6.5 × 10^2^	*E. cuniculi* II	1.7 × 10^2^	–	–
	Hrusice	53654	–	–	–	–	D	4.2 × 10^2^
	Jínošov	53685	–	–	–	–	CHALT-1	3.3 × 10^2^
	Kácov	53696	*C. sciurinum* VIIIc	3.9 × 10^2^	–	–	–	–
	Libeznice	53644	–	–	–	–	D	4.6 × 10^2^
	Maršovice	53662	–	–	–	–	D	3.8 × 10^2^
	Nesperská Lhota	53681	–	–	*E. cuniculi* I	1.1 × 10^1^	D	2.9 × 10^2^
	Netřebice	53640	–	–	–	–	D	6.1 × 10^2^
	Pacov	54620	*C. ubiquitum* XIId	2.7 × 10^2^	–	–	–	–
	Poděbrady	53645		–	*E. cuniculi* II	1.3 × 10^2^	–	–
	Podveky	53693	*C. sciurinum* VIIIc	1.0 × 10^2^	–	–	–	–
	Prostějov	57265	–	–	*E. cuniculi* II	2.7 × 10^2^	–	–
	Přední Lhota	53646	–	–	*E. cuniculi* II	4.2 × 10^2^	–	–
	Skalka	53704	*C. ubiquitum* XIId	9.8 × 10^3^	–	–	–	–
	Sulice	53665		–	*E. cuniculi* II	2.4 × 10^2^	–	–
	Vracovice	53708	–	–	–	–	CHN-F1	2.5 × 10^2^
	Záhornice	53638	–	–	*E. cuniculi* II	5.4 × 10^2^	–	–
	Vlašim	53673	–	–	*E. cuniculi* II	6.5 × 10^1^	–	–
		53676	–	–	*E. cuniculi* I	1.1 × 10^1^	–	–
		53697	–	–	*E. cuniculi* II	1.6 × 10^2^	–	–
		58354	–	–	*E. cuniculi* II	1.3 × 10^1^	–	–
SVK	Baloň	55632	–	–	*E. cuniculi* III	1.9 × 10^1^	–	–
		55635	*C. parvum* IIa	3.7 × 10^1^	–	–	–	–
	Dolné Lovčice	54915		–	–	–	C	5.7 × 10^2^
		54918	–	–	*E. hellem*	5.3 × 10^1^		–
		54922	–	–	–	–	C	3.1 × 10^2^
		54924	–	–	*E. cuniculi* II	1.1 × 10^1^	–	–
	Gabčíkovo	55770	–	–	*E. cuniculi* II	4.7 × 10^2^	–	–
	Krakovany	55828	–	–	*E. cuniculi* II	8.3 × 10^2^	–	–
	Kráľová pri Senci	55212	–	–	*E. cuniculi* II	9.2 × 10^1^	–	–
		55213	–	–	*E. cuniculi* I	6.7 × 10^1^	–	–
	Malé Dvorníky	55154	–	–	*E. cuniculi* II	3.3 × 10^3^	–	
	Okoč	54799	–	–	–	–	D	5.9 × 10^2^
		54802	deer mouse IV	6.9 × 10^1^	–	–	–	–
		54805	–	–	–	–	C	3.7 × 10^2^
	Prašník	55829	–	–	–		C	6.4 × 10^2^
	Skalica	57310	–	–	–		WildBoar3	9.2 × 10^1^
	Sklabiná	55274	–	–	*E. cuniculi* II	1.0 × 10^1^	D	1.3 × 10^2^
	Starý Háj	55268	–	–	*E. cuniculi* I	7.2 × 10^1^	–	–
	Topoľnica	55131	–	–	*E. cuniculi* II	4.1 × 10^2^	–	–
		55132	–	–	*E. cuniculi* II	2.3 × 10^3^	–	–
		55133	–	–	*E. cuniculi* II	1.1 × 10^2^	–	–
		55135	–	–	*E. cuniculi* II	8.5 × 10^2^	–	–
		55136	–	–	*E. cuniculi* II	3.4 × 10^2^	–	–
	Topoľníky	54833	*C. ubiquitum* XIIa	9.6 × 10^2^	*E. cuniculi* II	3.5 × 10^2^	–	–
	Trnava	54635	–	–	*E. cuniculi* II	7.5 × 10^3^	–	–
		54637	*C. ubiquitum* XIIa	5.2 × 10^3^	–	–	–	–
	Váh	55265	–	–	*E. cuniculi* II	3.6 × 10^2^	–	–
		55266	–	–	*E. cuniculi* II	1.8 × 10^3^	–	–
		55267	–	–	*E. cuniculi* I	3.7 × 10^1^	–	–
		55270	–	–	*E. cuniculi* I	5.9 × 10^1^	–	–
	Veľké Kosihy	54808	deer mouse IV	6.7 × 10^2^	–	–	–	–
		54809	*C. parvum* IIa	6.7 × 10^1^	–	–	–	–
		54813	deer mouse IV	8.7 × 10^1^	–	–	–	–
		54817	deer mouse IV	2.9 × 10^1^	–	–	–	–
	Vlčany	54926	*C. ubiquitum* XIIa	4.1 × 10^3^	–	–	–	–
		54929	–	–	*E. cuniculi* II	6.1 × 10^2^	–	–
		54933	deer mouse IV	8.2 × 10^2^	–	–	–	–
		54934	–	–	–	–	D	1.5 × 10^2^

**Figure 2:**
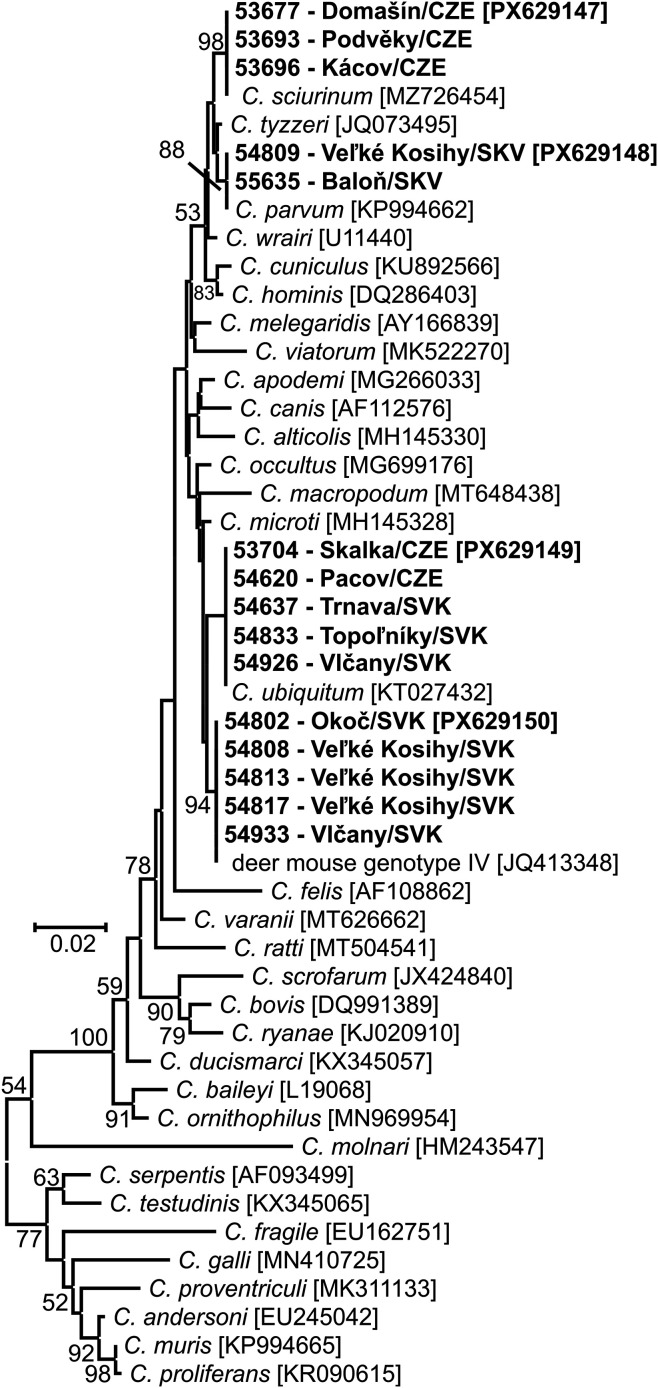
Cladogram illustrating the phylogenetic relationships of *Cryptosporidium* spp. detected in this study, together with other *Cryptosporidium* species and genotypes, based on a partial nucleotide sequence of the small subunit rDNA (18S rDNA) gene and constructed using the Maximum Likelihood method. Sequences obtained in this study are highlighted in bold. The GenBank accession number is given in square brackets. The locality and country of origin for each sample (CZE – Czechia, SVK – Slovakia) are indicated after the isolate identification number.

**Figure 3:**
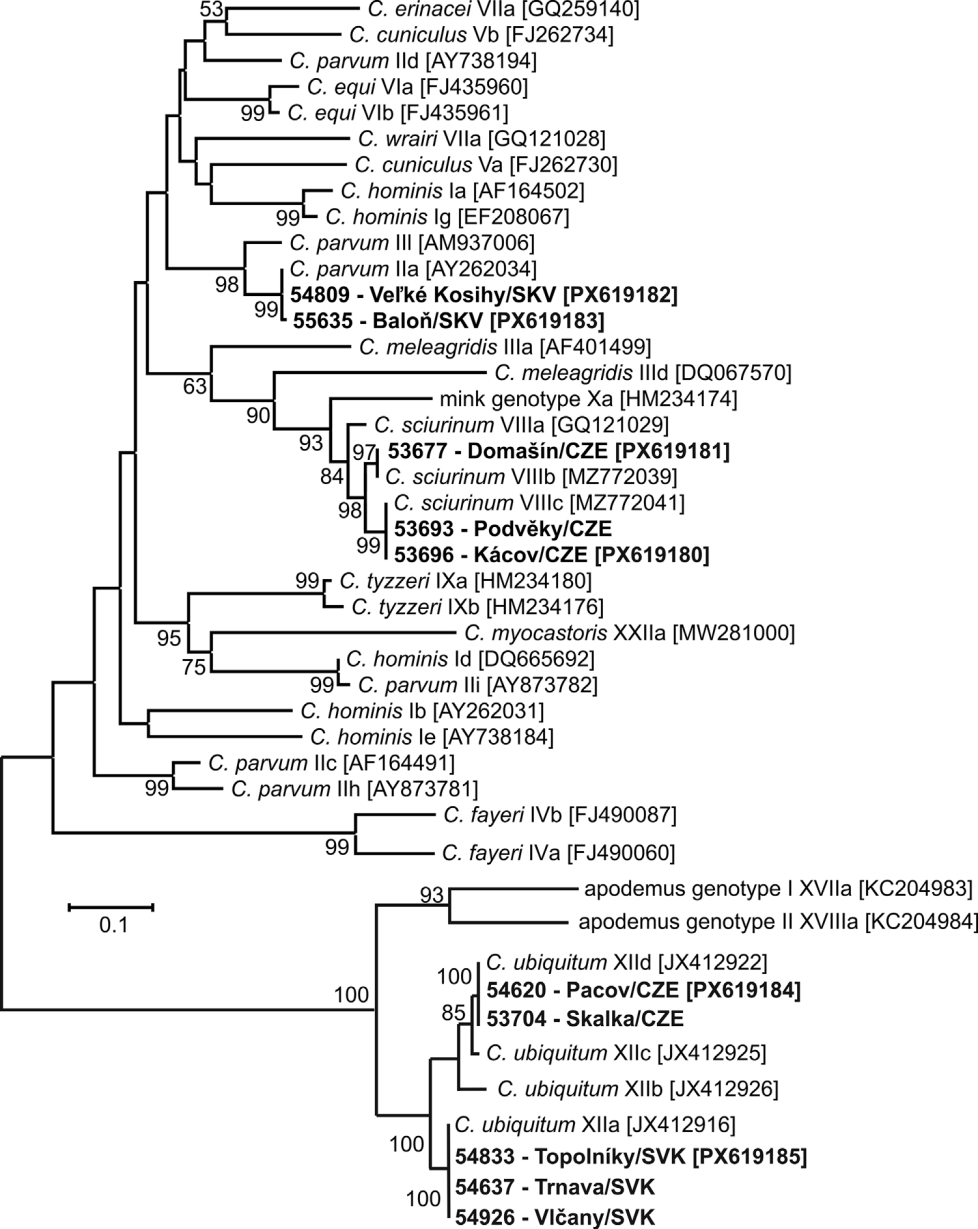
Cladogram illustrating the phylogenetic relationships of *Cryptosporidium* spp. detected in this study, together with other *Cryptosporidium* species and genotypes, based on a partial nucleotide sequence of the 60 kDa glycoprotein (*gp60*) gene and constructed using the Maximum Likelihood method. Sequences obtained in this study are highlighted in bold. The GenBank accession number is given in square brackets. The locality and country of origin for each sample (CZE – Czechia, SVK – Slovakia) are indicated after the isolate identification number.

*Cryptosporidium sciurinum* was detected only in Czechia, in three European brown hares from three different localities (Figs. 1 and 2). *Cryptosporidium parvum* was found in two individuals from different sites, while *Cryptosporidium* sp. deer mouse genotype IV was identified in five hares from three localities in Slovakia. *Cryptosporidium ubiquitum* was detected in hares from both Czechia and Slovakia (Figs. 1 and 2). Subsequent *gp60* subtyping revealed two *C. parvum* subtypes (IIaA15G1R1 and IIaA23G1R1), two *C. sciurinum* subtypes (VIIIcA10G2R1 [*n* = 2] and VIIIbA11G1R1 [*n* = 1]), and two *C. ubiquitum* subtypes (XIIa [*n* = 3] and XIId [*n* = 2]; Fig. 3). While subtype XIIa of *C. ubiquitum* originated solely from Slovakia, subtype XIId was found exclusively in Czechia (Fig. 3; Table 1). *Cryptosporidium sciurinum* subtype VIIIb was detected in a hare from a locality near the Czech–German border (Fig. 1). In contrast, subtype VIIIc was identified in two hares from two localities approximately 10 km apart in central Czechia. The infection intensity of *Cryptosporidium* spp. ranged from 2.9 × 10^1^ to 9.8 × 10^3^ OPG with the highest infection intensities observed in *C. ubiquitum* isolates (Table 1).

**Figure 4:**
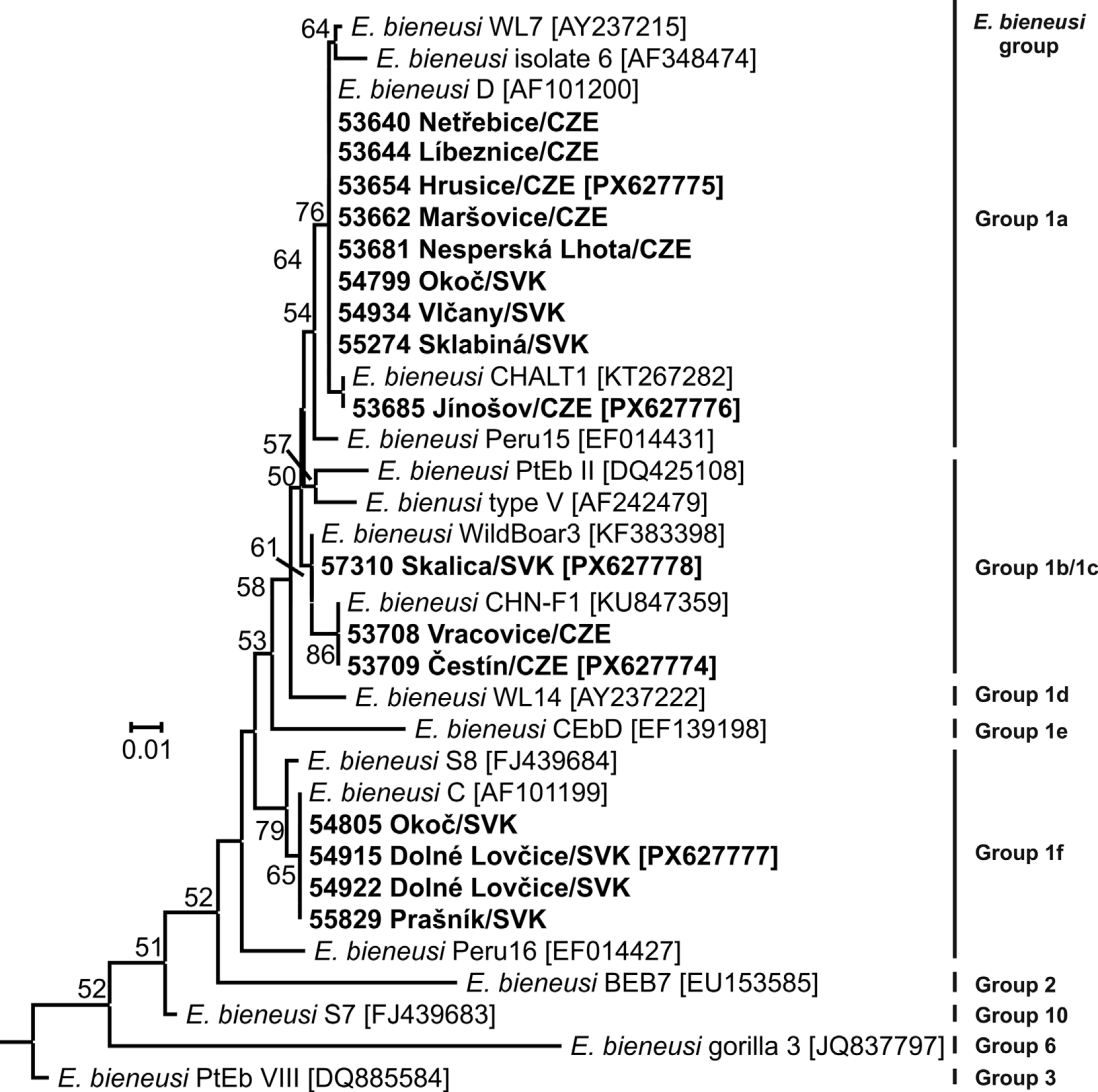
Cladogram illustrating the phylogenetic relationships of *Enterocytozoon bieneusi* detected in this study and other *E. bieneusi* genotypes, based on the complete nucleotide sequence of the internal transcribed spacer (ITS) region of the rRNA gene, constructed using the Maximum Likelihood method. Sequences obtained in this study are highlighted in bold. The GenBank accession number is provided in square brackets. The locality and country of origin of each sample (CZE – Czechia, SVK – Slovakia) are indicated after the isolate identification number. Detected genotypes were assigned to phylogenetic groups according to Li *et al.* [30] based on their host specificity.

In the analysed samples, five different genotypes of *Enterocytozoon bieneusi* were identified. Phylogenetic analyses revealed the presence of *E. bieneusi* genotype D (*n* = 8), genotype CHALT1 (*n* = 1), genotype WildBoar3 (*n* = 1), genotype CHN-F1 (*n* = 2), and genotype C (*n* = 4; Fig. 4). Genotypes C and WildBoar3 were detected exclusively in hares from Slovakia, while genotypes CHN-F1 and CHALT1 were found only in animals from Czechia Republic. The most frequently detected genotype D was identified in samples from both countries (Table 1; Fig. 4). The infection intensity of *Encephalitozoon* spp. ranged from 1.0 × 10^1^ to 7.5 × 10^3^ SPG (Table 1).

**Figure 5:**
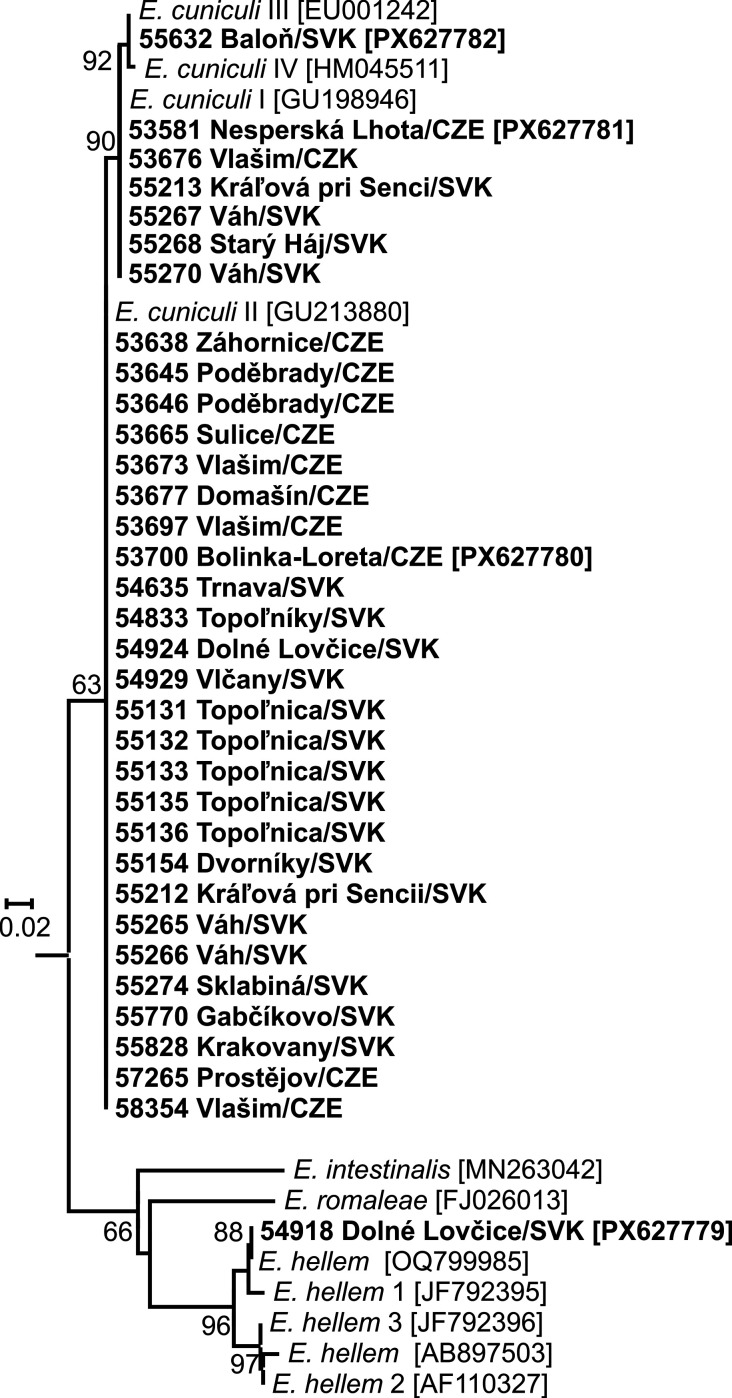
Cladogram illustrating the phylogenetic relationships of *Encephalitozoon* spp. detected in this study and other *Encephalitozoon* species and genotypes, based on the complete nucleotide sequence of the internal transcribed spacer (ITS) region of the rRNA gene, constructed using the Maximum Likelihood method. Sequences obtained in this study are highlighted in bold. The GenBank accession number is provided in square brackets. The locality and country of origin of each sample (CZE – Czechia, SVK – Slovakia) are indicated after the isolate identification number.

Phylogenetic analyses of *Encephalitozoon* spp. complete ITS sequences identified *E. cuniculi* genotype I (*n* = 6), *E. cuniculi* genotype II (*n* = 26), *E. cuniculi* genotype III (*n* = 1), and *E. hellem* (*n* = 1). The infection intensity of *E. bieneusi* genotypes ranged from 9.2 × 10^1^ to 6.4 × 10^2^ SPG. Genotypes D and C were detected most frequently and showed infection intensities similar to those of other genotypes detected sporadically. All sequences within each species/genotype were identical to each other and showed identity with sequences deposited in the GenBank database (Table 1; Fig. 5).

Animals kept in wildlife rescue centres were in good general condition, and faecal consistency was appropriate for their age and diet. No clinical signs commonly associated with *Cryptosporidium* or microsporidia infections, such as diarrhoea or neurological disorders, were observed. Hunted hares were also in good body condition, and all collected faecal samples were normally formed, with no evidence of diarrhoeal disease.

## Discussion

This study examined the occurrence and genetic diversity of *Cryptosporidium* spp., *G. intestinalis*, *Encephalitozoon* spp., and *E. bieneusi* in European brown hares in Czechia and Slovakia. We did not detect any hares infected with *G. intestinalis*, consistent with findings from Czechia, Croatia, and Italy, where no *Giardia* were found [6, 8, 33].

The low prevalence of *Cryptosporidium* detected in our study (4.1%) is consistent with previously published data on hare populations. Rego *et al.* (2022) identified *C. cuniculus* in 2% of Granada hare (*Lepus granatensis*) in Spain, while Li *et al.* reported *Cryptosporidium* sp. skunk genotype in 7.7% of snowshoe hares (*Lepus americanus*) in North America. In European brown hares, *Cryptosporidium* has been reported only by Lukešová *et al.* (2012), who microscopically detected five positive cases out of 360 examined individuals, although the isolates were not genotyped. The low microscopic positivity observed in these studies aligns with our results, in which oocysts were identified microscopically in only three out of 370 European brown hares. For the first time, we reported the occurrence of four distinct *Cryptosporidium* species/genotypes in European brown hares. The detected species *C. ubiquitum* and *C. parvum* are characterised by broad host specificity, so their presence in hares is not unexpected. Subtyping of both species at the *gp60* locus revealed the presence of subtypes XIIa and XIId in *C. ubiquitum* and subtype IIa in *C. parvum*, all of which are commonly found in wild and domestic ruminants [27, 45]. These hosts show considerable ecological overlap with hares, sharing the same permanent grasslands, field margins and pastures [53]. Habitat overlap of this type increases the likelihood of indirect contact and may facilitate the environmental transmission of these *Cryptosporidium* species. In contrast, *C. sciurinum*, another species detected in our study, is host-specific for squirrels, with which hares have only limited ecological overlap. Because all hares positive for *C. sciurinum* originated from rescue centres, where this species had previously been detected in squirrels, environmental contamination or pseudoparasitism resulting from the passive passage of oocysts through the gastrointestinal tract cannot be completely ruled out. However, faecal samples from rescue animals were collected immediately upon arrival at the rescue centres, or within 24 h, before transfer to animal holding facilities, which substantially reduces the likelihood of contamination occurring within the centres. In addition, the three *C. sciurinum*-positive hares originated from three different localities and two independent rescue centres, and two distinct *gp60* subtypes (VIIIb and VIIIc) were identified. Therefore, the present findings should be interpreted with caution. Nevertheless, natural transmission in the wild cannot be fully ruled out, as *C. sciurinum* is commonly found in free-living squirrels in Europe [22, 42, 43]. Both *gp60 C. sciurinum* subtypes detected in hares in this study have previously been identified in squirrels within Czechia [43]. The detection of *C. sciurinum* DNA in European brown hares suggests that this species may occasionally infect or pass through this host, but further studies are needed to confirm true infection and determine its host range. The last detected *Cryptosporidium*, the deer mouse genotype IV, represents an unexpected finding. This genotype has so far been reported only in the deer mouse (*Peromyscus maniculatus*) in the United States and Canada [31, 32, 47, 54]. Although pseudoparasitism cannot be entirely ruled out, the repeated detection of deer mouse genotype IV in several hares from different localities, together with the observed infection intensities, supports the hypothesis of true infection rather than passive passage of oocysts through the gastrointestinal tract. However, even if these findings represent pseudoparasitism, the presence of this genotype in Central Europe remains unexplained. Unlike *C. mortiferum*, whose introduction into Europe has been linked to the spread of grey squirrels from North America [42, 59], there is currently no information on the origin, transmission routes, or potential reservoir hosts of deer mouse genotype IV in Europe. Whether the detected hares were true hosts or merely accidental carriers, the present findings indicate that this genotype circulates in the Central European environment and requires further investigation. Interestingly, the species composition of *Cryptosporidium* detected in European brown hares differs substantially from that reported in rabbits. In both domestic and wild rabbits, *C. cuniculus* is considered the predominant species and has been reported worldwide [27, 45]. In contrast, *C. cuniculus* has not been detected in European brown hares to date, including in the present study. This observation may suggest differences in host susceptibility among lagomorph species. However, given the limited number of molecular studies conducted in hares, the apparent absence of *C. cuniculus* should be interpreted with caution.

The number of studies and confirmed cases of parasites of the genus *Encephalitozoon* in hares is limited. Only two studies have reported *Encephalitozoon* infections in hares based on PCR detection. De Bosschere *et al.* [10] identified *E. intestinalis* and *E. hellem* in a single European brown hare, while Martínez-Padilla *et al.* [34] detected *E. intestinalis* in three Iberian hares. Other studies have reported only the presence of specific antibodies in European brown hares [3, 38]. The prevalences reported in these studies (0.5–3.8%) were lower than in our study (8.1%). However, this difference may be attributable to the larger number of examined animals in our dataset. Another difference lies in the species composition of microsporidia of the genus *Encephalitozoon*. In our study, *E. cuniculi* was the dominant species, with genotype II being the most frequently detected, followed by genotypes I and III. Genotype II is a common genotype reported in both domestic and wild animals, whereas genotypes I and III occur less frequently and are detected only in a small proportion of cases [19]. Only one *E. hellem* isolate was detected in our study, a finding that is consistent with the observations of Martínez-Padilla *et al.* [34], as this species is predominantly associated with avian hosts and is only sporadically reported in mammals. Interestingly, the detected isolate does not match any sequence published to date. It most likely represents a novel subtype within the species *E. hellem*. The predominance of *E. cuniculi* observed in the present study is consistent with previous findings in rabbits (*Oryctolagus cuniculus*). Molecular investigations in rabbits have reported prevalences ranging from (4.0–60%) in wild, domestic, and farmed rabbits by PCR [5, 7, 11, 34]. Higher prevalences have been reported when urine or tissue samples were analysed [5, 7, 64]. Although *E. cuniculi* genotype I is commonly referred to as the rabbit genotype, genotype II has also been reported relatively frequently in rabbit populations [5, 11].

In contrast to previous studies where no *E. bieneusi* in hares was detected, we found *E. bieneusi* in 4% of animals [34, 44]. The dominant genotypes were C and D, both of which have previously been reported in a wide range of domestic and wild animals as well as in humans [23, 49]. Their presence in the examined hares is therefore not unexpected, as environmental contamination with these genotypes is likely common due to their low host specificity. The detection of the wild-boar genotype, originally identified in wild boar populations, is also unsurprising given the increasing overlap of wildlife habitats [36]. The identification of two additional genotypes, CHN-F1 and CHALT1, previously reported in raccoon dogs and foxes, and tigers in China, respectively, suggests that transmission to hares may occur through contact with wild carnivores [28, 63]. Compared with rabbits, for which numerous *E. bieneusi* genotypes have been reported worldwide, the genotype diversity observed in European brown hares appears relatively limited. However, previous studies in rabbits have demonstrated considerable geographical variation in genotype composition, suggesting that local epidemiological conditions may influence the observed diversity [5, 11]. As molecular data on *E. bieneusi* in hares remain scarce, caution is warranted when comparing genotype richness between the two lagomorph hosts, and further studies from different geographic regions will be necessary before broader conclusions can be drawn.

Several of the species and genotypes identified in this study have recognised zoonotic potential. Both *C. parvum* and *C. ubiquitum*, including the detected subtype families IIa, XIIa, and XIId, have previously been reported in humans. Similarly, *E. cuniculi* genotypes I–III and *E. bieneusi* genotypes C, D, CHN-F1, WildBoar3, and CHALT1, all belonging to phylogenetic group 1, which comprises most zoonotic genotypes [29, 30, 49]. These findings suggest that European brown hares may contribute to the environmental circulation of zoonotic protists.

*Cryptosporidium* spp. and microsporidia are recognised enteric pathogens capable of causing clinical disease in a wide range of hosts. *Cryptosporidium* and *E. bieneusi* infections are mostly associated with diarrhoea and gastrointestinal disorders, particularly in young, immunocompromised, or stressed individuals [45]. In contrast, infections caused by *Encephalitozoon* spp. may affect not only the gastrointestinal tract but also other organs, and in severe cases can be associated with neurological manifestations, renal lesions, or ocular disease [49]. No clinical signs of disease associated with the detected parasites were observed in any of the animals, especially those housed in rescue centres. Based on these findings, it can be inferred that the infections were asymptomatic and had no apparent impact on the animals’ health. These results are consistent with numerous studies showing that wild animals are commonly infected with *Cryptosporidium* spp. and microsporidia, which, however, typically do not cause clinically significant disease in these hosts [27, 49].

## Conclusions

To date, this study provides the most comprehensive molecular analysis to date of *Cryptosporidium* spp., *Encephalitozoon* spp., and *E. bieneusi* in European brown hares from Czechia and Slovakia. We identified four *Cryptosporidium* spp., including the deer mouse genotype IV, which we reported here for the first time in Europe. Within the genus *Encephalitozoon*, *E. cuniculi* genotype II was predominant. Unlike previous studies, we also detected five different genotypes of *E. bieneusi*. The higher prevalence and diversity observed in this study are likely due to the larger number of animals examined and the use of highly sensitive molecular methods. Overall, our results significantly expand current knowledge of parasitic protist infections in hares.

## Supplementary material

*Table S1*. Detailed information on sampling localities, the number of samples collected at each locality, and all available metadata for individual animals, including age and origin. *Table S2*. Primers and probes sequences used for the detection of *Cryptosporidium* spp., *Encephalitopzoon* spp. and *Giardia intestinalis*. The supplementary material of this article is available at https://www.parasite-journal.org/10.1051/parasite/2026042/olm.
