# Supplementary material for: Genetic characterisation of Cryptosporidium spp., Encephalitozoon spp., and Enterocytozoon bieneusi in European brown hare (Lepus europaeus) in Central Europe
Source: Parasite. 2026 Aug 6;33:43. doi: 10.1051/parasite/2026042 (PMC13446113; doi:10.1051/parasite/2026042)
Supplement: Supplementary file 1 [file parasite-33-43-s2.pdf]

Table S2. Primers and probes sequences used for the detection of *Cryptosporidium* spp., *Encephalitopzoon* spp. and *Giardia intestinalis*.

Targeted parasite	Primers and probes sequences (5'-3')		Reference
Cryptosporidium	forward	ATGACGGGTAACGGGGAAT	[18]
	reverse	CCAATTACAAAACCAAAAAGTCC	
	probe	FAM-CGCGCCTGCTGCCTTCCTTAGATG- BHQ	
Encephalitozoon	forward	GTC CGT TAT GCC CTG AGA T	[60]
	reverse	ACAGCAGCCATGTTACGACT	
	probe 1	FAM-GCCCGTCGCTATCTAAGATGACGCA- BHQ	
	probe 2	HEX-TGGACGAAGATTGGAAGGTCTGAGTC-BHQ	
Giardia intestinalis	forward	CGGACACCGCTGGCAA	[25]
	reverse	CTGCGTCACGCTGCTCG	
	probe	FAM-GCCCGCCCTTGCGCGCACG-BHQ	
